# Magnetic Resonance Imaging in the Assessment of the Risk of Sudden Death in Cardiac Sarcoidosis: What Is Extensive or Significant Late Gadolinium Enhancement?

**DOI:** 10.1161/CIRCEP.124.013239

**Published:** 2024-12-20

**Authors:** Pauli Pöyhönen, Jukka Lehtonen, Suvi Syväranta, Diana Velikanova, Henriikka Mälkönen, Piia Simonen, Hanna-Kaisa Nordenswan, Valtteri Uusitalo, Tapani Vihinen, Kari Kaikkonen, Petri Haataja, Tuomas Kerola, Tuomas T. Rissanen, Ville Vepsäläinen, Aleksi Alatalo, Päivi Pietilä-Effati, Markku Kupari

**Affiliations:** Heart and Lung Center, Helsinki University Hospital and University of Helsinki, Finland (P.P., J.L., D.V., H.M., P.S., H.-K.N., M.K.); Radiology, Helsinki University Hospital and University of Helsinki, Finland (P.P., S.S., V.U.); Clinical Physiology and Nuclear Medicine, Helsinki University Hospital and University of Helsinki, Finland (V.U.).; Heart Center, Turku University Hospital, Finland (T.V.).; Medical Research Center Oulu, University and University Hospital of Oulu, Finland (K.K.).; Heart Hospital, Tampere University Hospital, Finland (P.H.).; Department of Internal Medicine, Päijät-Häme Central Hospital, Lahti, Finland (T.K.).; Heart Center, North Karelia Central Hospital, Joensuu, Finland (T.T.R.).; Institute of Clinical Medicine, University of Eastern Finland, Kuopio, Finland (T.T.R.).; Heart Centre, Kuopio University Hospital, Finland (V.V.).; South Ostrobothnia Central Hospital, Seinäjoki, Finland (A.A.).; Vaasa Central Hospital, Finland (P.P.-E.).

**Keywords:** contrast media, gadolinium, incidence, magnetic resonance imaging, sarcoidosis, sudden death

## Abstract

**BACKGROUND:**

Cardiac sarcoidosis involves a significant but difficult-to-define risk of sudden cardiac death (SCD). Current guidelines recommend consideration of an implantable cardioverter defibrillator for patients with extensive or significant myocardial late gadolinium enhancement (LGE) on cardiac magnetic resonance imaging. However, extensive/significant LGE is not defined.

**METHODS:**

A nationwide cardiac sarcoidosis registry was screened for patients entered before 2020 with cardiac magnetic resonance imaging done before or <3 months after diagnosis. Available studies were re-analyzed for LGE mass as a percentage of left ventricular (LV) mass and the number of LGE-positive LV segments in a 17-segment model. The occurrence of fatal or aborted SCD and ventricular tachycardia (VT) prompting therapy was recorded until the end of 2020 and subjected to cumulative incidence analyses, including competing events (LV assist device implantations, heart transplantations, and fatalities other than SCD). The predictors of SCD/VT were assessed using Fine and Gray modeling and time-dependent receiver operating characteristic analysis.

**RESULTS:**

Altogether, 305 patients (66% women, median age 51) with clinically manifest, definite (45%) or probable cardiac sarcoidosis (55%) were analyzed. On follow-up (median, 4.0 years), 21 SCDs, 60 VTs, and 14 competing events were noted. Both LGE mass and the number of LGE segments predicted the composite of SCD/VT (*P*<0.001), with receiver operating characteristic analyses identifying LGE mass ≥9.9% and ≥6 LGE segments as discriminative thresholds. At presentation, 70 patients were free of class I and class IIa implantable cardioverter defibrillator indications unrelated to LGE. Their 5-year rate of SCD/VT was 6.3% (0.0–14.8%) with LGE mass <9.9% versus 21.5% (6.5–36.6%) with higher LGE mass, and 6.9% (0.0–16.3%) with <6 LGE segments versus 20.5% (5.9–35.2%) with ≥6 segments.

**CONCLUSIONS:**

In cardiac sarcoidosis, myocardial LGE making up ≥9.9% of LV mass or affecting ≥6 LV segments may suggest prognostically significant LV involvement and a high risk of SCD. However, prospective validation of the thresholds is needed.

**WHAT IS KNOWN?**

- Clinically manifest cardiac sarcoidosis involves a propensity for ventricular tachyarrhythmias and a consequent risk of sudden cardiac death that approximates 10% in 5 years, but is difficult-to-define individually.
- Current guidelines for cardiac sarcoidosis recommend a primary prevention implantable cardioverter defibrillator if cardiac magnetic resonance imaging shows extensive or significant myocardial late gadolinium enhancement, without specifying what constitutes extensive/significant late gadolinium enhancement.

**WHAT THE STUDY ADDS**

- Late gadolinium enhancement mass ≥9.9% of left ventricular mass or the presence of ≥6 late gadolinium enhancement-positive left ventricular segments on cardiac magnetic resonance imaging could be considered prognostically extensive left ventricular involvement in assessing the risk of sudden cardiac death in cardiac sarcoidosis.

Cardiac sarcoidosis (CS) is a rare inflammatory cardiomyopathy resulting from noncaseating granulomas injuring and scarring the myocardium. When clinically manifest, CS presents commonly as high-grade atrioventricular block, congestive heart failure, or fast arrhythmias including sustained ventricular tachycardia (VT) and ventricular fibrillation (VF) causing cardiac arrest.^1,2^ Life-threatening arrhythmias prevail as serious events during treatment and follow-up because neither immunosuppression, antiarrhythmic drugs, nor catheter ablation can eliminate the arrhythmogenicity of CS.^1,3^ The risk of sudden cardiac death (SCD) approximates 10% at 5 years from disease presentation,^4^ with the combined rate of SCD and sustained VT approaching 25%.^4,5^ An implantable cardioverter defibrillator (ICD) is a potential life-saver in CS,^6,7^ being indicated for secondary prevention in survivors of cardiac arrest and patients with documented sustained VT.^7–9^ Implantations for primary prevention are more challenging because there exist neither established risk assessment algorithms nor ICD trials in CS. Guidelines for the treatment of ventricular arrhythmias and prevention of SCD, issued by the American College of Cardiology, American Heart Association (AHA), and Heart Rhythm Society (HRS),^8^ and by the European Society of Cardiology (ESC),^9^ give a strong recommendation (class I) for an ICD if left ventricular ejection fraction (LVEF) is ≤35% and modest recommendations (class IIa) if LVEF is >35% but there is either an indication for permanent pacing,^8,9^ inducible VT,^8,9^ a history of arrhythmogenic syncope,^8^ or the presence of late gadolinium enhancement (LGE) on cardiac magnetic resonance imaging (CMRI) suggesting extensive^8^ or significant^9^ LV scar. Though endorsing LGE imaging for risk assessment in CS, neither guideline defines what extensive or significant LGE is, thereby providing limited help for clinical decision-making. Pending more specific definitions, we analyzed CMRI studies of >300 patients with biopsy-proven CS for the capacity of detailed LGE findings to predict life-threatening arrhythmias and SCD. The data we present here may help translate extensive/significant LGE into numbers for clinical work.

## Methods

Individual-level data cannot be shared openly or made available to other researchers for purposes of reproducing the results due to restrictions by patient consent. The corresponding author (P.P.) had full access to all data and takes responsibility for its integrity and data analysis.

### Study Population

At the end of 2019, the ongoing nationwide registry of Myocardial Inflammatory Diseases in Finland (MIDFIN)^2,4,10^ included 512 patients with a lifetime diagnosis of CS by the HRS criteria.^7^ The patients had been collected retrospectively from the late 1980s but mainly prospectively since 2009. We considered for the present work all patients with a CMRI study done before the diagnosis of CS or <3 months thereafter. Altogether, 349 patients were eligible, with 305 having studies that were available for reanalysis; they constitute our study population. The patients had been examined and entered into the MIDFIN registry between October 2003 and December 2019. The present work continues our research on SCD and life-threatening arrhythmias in CS,^2,4^ adding a comprehensive analysis of prognostic factors with a focus on CMRI and details of LGE imaging.

### Data Collection

The baseline characteristics of the patients were retrieved from the MIDFIN registry, the framework of which has been described in our previous publications.^2,4,10^ Patients’ demographics, presenting cardiac manifestations, comorbidities, and the results of conventional diagnostic studies, including echocardiography, biopsies, and routine laboratory examinations, were extracted from the database of the registry. The details of medical, surgical, and catheter- or device-based therapies were also collected from the registry, with re-checks of hospital documents, if necessary, as were the dates and types of cardiac and noncardiac adverse events occurring during follow-up. All CMRI examinations done before or <3 months following CS diagnosis were acquired for reanalysis in the MIDFIN core center (Helsinki University Hospital), as detailed below. Available 18F-fluorodeoxyglucose (FDG) positron emission tomography (PET) scans taken before diagnosis were also re-reviewed in the core center, with the presence of abnormal cardiac FDG uptake (focal or focal-on-diffuse) being incorporated into the present dataset as a sign of active myocardial inflammation. The analysis of the PET scans was done by an expert in nuclear medicine (V.U.) masked to clinical and CMRI data.

### CMRI Studies

CMRI examinations were conducted on 1.5T or 3T CMRI scanners in the participating hospitals using phased-array receiver coils and standard protocols^11,12^ according to contemporaneous local routines. Breath-hold cine studies were performed using electrocardiographically gated steady-state free precession imaging. To assess left and right ventricular volumes and ejection fraction, cine images were obtained in long-axis (2-, 3-, and 4-chamber views) and short-axis planes covering both ventricles (typical slice thickness 6–8 mm, interslice gap 20%). LGE imaging was performed 10 to 15 minutes after an intravenous injection of contrast agent (0.15 mmol/kg) using an inversion-recovery gradient-echo sequence in views identical to cine imaging. Twenty-two patients (7%) had an intracardiac device (pacemaker/ICD) during imaging.

### CMRI Analyses

**Figure 1.**
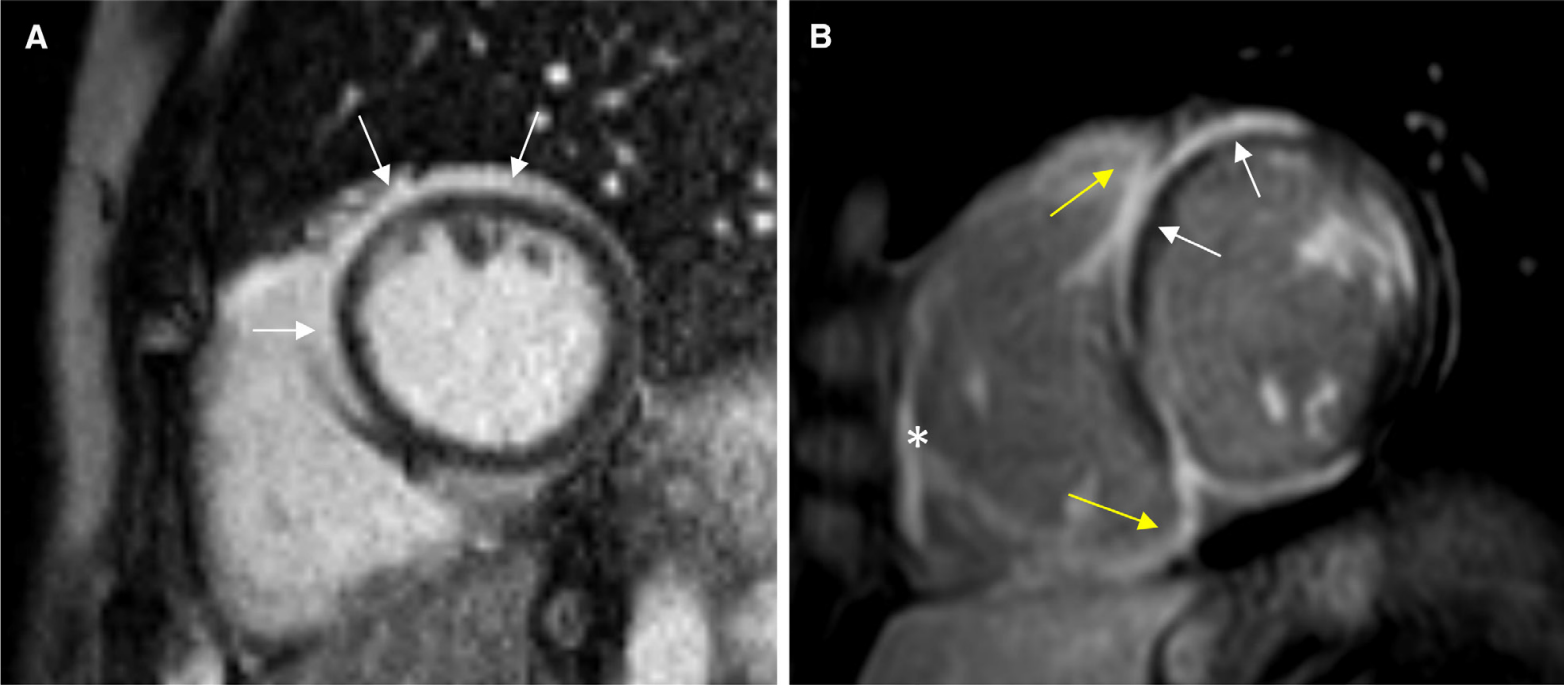
**Contrast-enhanced magnetic resonance imaging phenotypes of cardiac sarcoidosis featuring the distribution of late gadolinium enhancement (LGE).** The images present short-axis views of both ventricles. **A**, Demonstrates ring-like LGE^17,18^ with continuous subepicardial enhancement in the basal anterior and anteroseptal left ventricular segments (arrows), extending into parts of the inferoseptal and anterolateral segments. **B**, Shows right ventricular free-wall LGE (asterisk), the hook-sign LGE at septal insertions (yellow arrows), as well as septal and subepicardial LGE (white arrows), demonstrating multifocal myocardial involvement and thus all 4 features of pathology-frequent LGE.^20.^

The studies that were available for re–evaluation and passed an initial quality check were analyzed in random order by a cardiologist with expertise in CMRI (P.P.), blinded to all clinical data. Left and right ventricular volumes and LV mass were determined with standard protocols,^13^ including papillary muscles and outflow tract in the LV volume. The presence of LGE was assessed visually and the number of positive LV segments was counted according to the AHA 17-segment model.^14^ The extent of LGE as a percentage of LV mass was assessed using the full-width at half-maximum method.^15,16^ The LGE pattern was classified as subendocardial, mid-myocardial, subepicardial, or transmural in each segment. The presence of subepicardial or mid-myocardial LGE in at least 3 contiguous LV segments in the same short-axis slice was recorded as the ring-like LGE,^17,18^ and a prominent involvement of ventricular insertion points with contiguous extension of LGE from the septum into the right ventricular (RV) free wall was noted as the hook sign (or hug sign).^19^ Furthermore, pathology-frequent distribution of LGE^20^ was identified as the presence of either (1) subepicardial LGE (including RV-side of septum), (2) multifocal LV LGE, (3) septal LGE, or (4) RV free-wall LGE, with the score of the criteria (0–4) recorded for each patient as previously described.^20^ Figure 1 exemplifies the different LGE phenotypes identified and recorded for prognostic analyses. Image analysis was performed by QMass MR Software (version 8.1, Medis Medical Imaging Systems, Leiden, the Netherlands).

To assess the interobserver reproducibility of the LGE analyses, the studies of 20 random subjects were read independently by a cardiothoracic radiologist (S.S.) using the methods described above. Bland-Altman analysis^21^ showed a mean difference of 0.9±2.0 in the number of LGE-positive segments (*P*=0.074) and −1.1±3.8% in LGE mass (*P*=0.227). The intercorrelation coefficient (2-way, absolute agreement) was 0.80 for the number of LV segments and 0.91 for LGE mass. Cohen κ statistic was 0.60 for hook sign, 0.89 for ring sign, and 0.71 for the presence of RV free-wall LGE. Comparable intraobserver analyses showed a mean difference of 0.4±1.5 in the number of LGE-positive segments (*P*=0.309) and 1.3±3.2% in LGE mass (*P*=0.085). The intercorrelation coefficient (2-way, absolute agreement) was 0.88 for the number of LV segments and 0.92 for LGE mass. Cohen κ statistic was 0.80 for hook sign, 0.71 for ring sign, and 0.80 for the presence of RV free-wall LGE.

### Definition of Outcome Events

The main outcome end point was the first occurrence of either SCD, aborted SCD, or sustained VT converted by either external cardioversion/defibrillation, ICD therapy, or in-hospital amiodarone infusion. Separate analyses were made of SCD, fatal or aborted, as the outcome end point, ignoring VTs. Aborted SCD was defined as a documented episode of VF terminated successfully either by an ICD or by external defibrillation during resuscitation for cardiac arrest. Cardiac transplantation, implantation of a left ventricular assist device, and death attributable to either noncardiac causes or terminal heart failure were analyzed as competing events. The dates and characters of the events were identified and ascertained by the cardiologists of the MIDFIN research network (the present authors in their respective hospitals) from medical records, 12-lead electrocardiograms, and ICD reports. The causes of death were determined by scrutiny of medical records and findings at autopsy. The follow-up lasted until December 31, 2020, assuring a minimum of 1-year surveillance for each patient. No patients were lost to follow-up. Right censoring was defined as no event or competing event before the end of follow-up.

### Ethical Approvals

The MIDFIN registry study was approved by the national ethical review board in 2009 (STM(Ministry of Social Affairs and Welfare)/1219/2009). All involved centers granted approval for the study. Each patient alive at the time of recruitment gave written informed consent. The reanalyzes of CMRI studies were covered by local ethical board approvals (HUS(Helsinki University Hospital)/144/2020, HUS/54/2019, and HUS/27/2012).

### Statistical Analyses

Continuous variables are presented as mean±SD for normally distributed data and as median (interquartile range) for skewed data. Categorical variables are presented as frequencies (%). Group comparisons were performed with one-way analysis of variance, Kruskal-Wallis test, χ^2^ test, or Fisher exact test, as appropriate. Follow-up times were calculated from the date of the CMRI study. Cause-specific cumulative incidence analysis^22^ was used to calculate the incidence estimates with 95% CIs for the end point events; the Gray test^23^ was used for comparisons between groups. The CIs for cumulative incidence estimates were calculated by estimate ±1.96× the square root of the variance. Events per exposure time (100 patient-years) and their Poisson 95% CI were also calculated. The Fine and Gray model was used to calculate subdistribution hazard ratios with 95% CIs.^24^ The proportionality of hazards was ascertained by Schoenfeld residuals, and significant multicollinearity was excluded by variance inflation factor values. Only cases with complete data were included in the analyses. Cumulative time-dependent receiver operating characteristic analyses incorporating competing events were performed to study the predictive capacity of baseline CMRI variables and to search for cutoffs for continuous variables.^25^ In these analyses, controls were defined as survivors free of arrhythmic events or subjects with a competing event before the follow-up time of interest. Cutoff values were chosen using the Youden index with equal weight on sensitivity and specificity. Values of *P*<0.05 were considered statistically significant. Analyses were performed with SPSS-29 (SPSS, Inc, Chicago, IL) and R (version 4.1.2, The R Foundation, Vienna, Austria).

## Results

### Clinical and CMRI Characteristics of the Study Population

Altogether 305 patients (median age, 51; 66% women) had CMRI studies that were available for reanalysis and dated before the diagnosis of CS or <3 months thereafter. The diagnosis was definite by myocardial biopsy in 136 patients (45%), the rest having probable CS by the HRS diagnostic scheme,^7^ with sarcoidosis histology proven from extracardiac tissues or lymph nodes. All patients were white Northern Europeans. Table 1 presents the patients’ clinical characteristics at presentation stratified by the occurrence of outcome events. High-grade atrioventricular block was the leading initial cardiac manifestation (45%), with serious ventricular tachyarrhythmia (sustained VT or aborted SCD) being the second most common mode of presentation (20%). The median echocardiographic LVEF was 55% at presentation. Abnormal cardiac ^18^F-FDG uptake on PET scans and elevated circulating cardiac troponins were observed as signs of active inflammation in 92% and 49% of patients undergoing these examinations, respectively. Less than 20% had a history of preexisting sarcoidosis on admission.

**Table 1.**
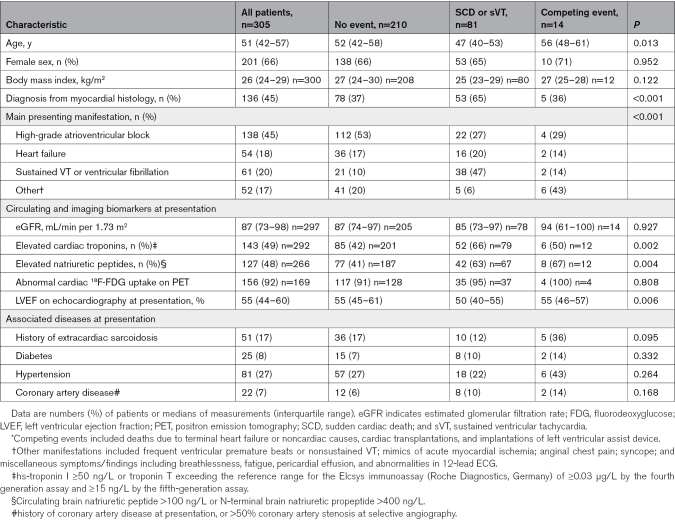
Patient Characteristics at Presentation of Cardiac Sarcoidosis According to the Occurrence of Arrhythmic and Competing Events^*^ on Follow-Up

Table 2 summarizes the findings on CMRI and shows that nearly all patients (96%) had LGE on the LV myocardium. RV free-wall LGE was found in 39% of patients, with all but 1 individual having biventricular involvement. The median LVEF was slightly reduced at 48%, the median number of LGE-positive segments was 6, and the median LGE mass was 13.7%. The ring-like LGE, the hook sign, and LGE with ≥1 pathology-frequent feature (see Methods section) were present in 40%, 31%, and 99% of LGE-positive patients, respectively. Comparisons across the 3 groups (Table 2) showed differences in CMRI variables indicating most marked myocardial involvement and dysfunction in patients with SCD or VT on follow-up.

**Table 2.**
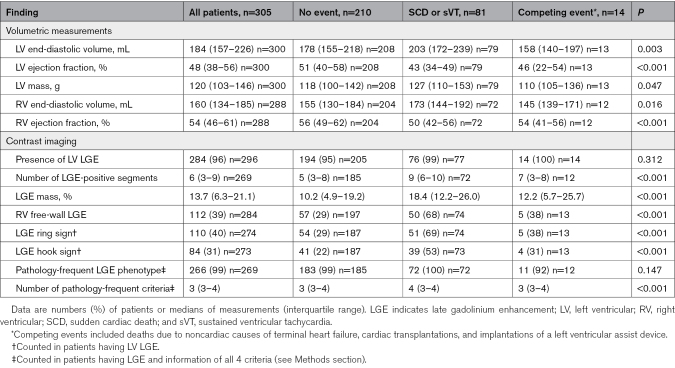
Findings on Cardiac Magnetic Resonance Imaging According to the Occurrence of Follow-Up Events

### Treatment in Brief

Of the entire cohort, 98% (299/305 patients) received corticosteroid-based, tiered immunosuppression with azathioprine (32%), methotrexate (11%), mycophenolate (7%), cyclosporin (6%), and infliximab (5%) as adjunctive immunomodulators. Furthermore, 95% of patients were on β-adrenergic blockers, 67% on angiotensin-converting enzyme inhibitors/angiotensin receptor blockers, and 30% on mineralocorticoid receptor antagonists during follow-up. By the end of follow-up, 19 (6%) had undergone VT ablation, 242 (79%) had received an ICD, and 24 patients (8%) had a permanent pacemaker or cardiac resynchronization therapy device without defibrillating capacity.

### Events and Their Predictors on Follow-Up

During the median follow-up of 4.0 years (2.0–6.4 years), 81 patients experienced either SCD (n=21, 2 fatal) or VT prompting treatment (n=60) as their first event, and 14 had competing events consisting of 9 deaths, 3 transplantations, and 2 left ventricular assist device implantations. Excluding VTs from the analysis, 32 patients suffered a SCD (2 fatal) and 22 experienced a competing event over the median follow-up of 4.9 (2.8–6.9) years. The cause-specific 5-year incidence (95% CI) in the entire cohort was 26% (21–32%) for the composite of SCD/VT and 10% (6–14%) for SCD, with 10-year estimates being 35% (27–42%) for SCD/VT and 16% (10–21%) for SCD alone.

The predictors of the composite of SCD/VT by univariable Fine and Gray analyses are shown in Table 3. Younger age, lower body mass index, definite CS diagnosis, main presenting manifestation, lower LVEF on echocardiography, and elevated concentrations of troponins and natriuretic peptides at presentation were predictive of SCD/VT. Sex, renal function, and abnormal cardiac ^18^F-FDG uptake were nonpredictive (Table 3), as were all associated diseases (data not shown). Most CMRI variables were statistically highly significant predictors of SCD/VT. Neither the mere presence of LV LGE nor the pathology-frequent LGE phenotype were predictive, however, both being nearly ubiquitous in the subgroups with and without future events. The count of the pathology-frequent LGE features had, instead, prognostic significance. A multivariable model incorporating the number of LGE-positive LV segments with the key non-CMRI predictors is shown in Table 4. The model highlights the presentation with ventricular tachyarrhythmia as the strongest independent predictor of future SCD/VT, with the number of LGE-positive segments reaching, and elevation of cardiac troponins approaching independent statistical significance. Replacing the count of LGE-positive segments in the multivariable model with the other LGE variables, 1 at a time, yielded adjusted subdistribution hazard ratios of 1.10 (0.96–1.25, *P*=0.160) for the LGE mass (per +5%), 2.63 (1.47–4.73, *P*=0.001) for the ring-like LGE, 2.14 (1.27–3.60, *P*=0.004) for the hook sign, 2.63 (1.56–4.44, *P*<0.001) for the RV free-wall LGE, and 2.10 (1.29–3.42, *P*=0.003) for the count of the pathology-frequent LGE criteria (per +1 criterion).

**Table 3.**
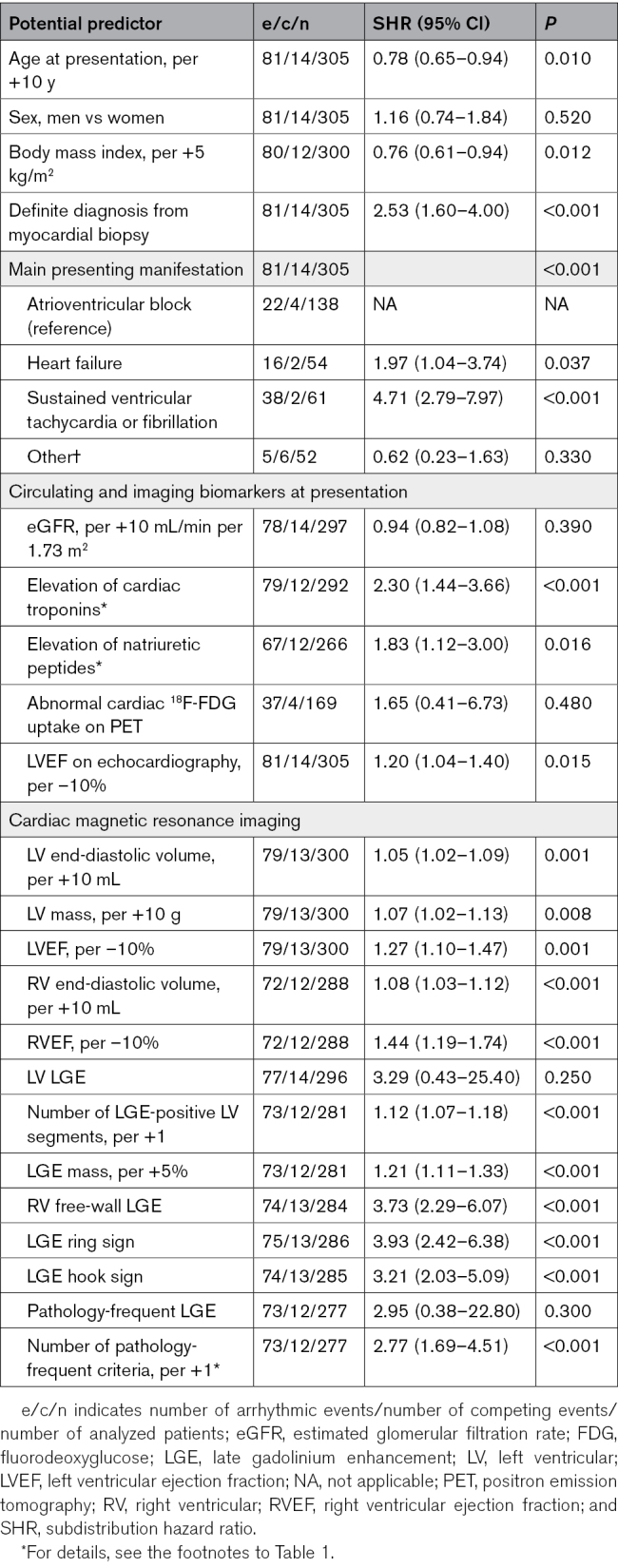
Predictors of Life-Threatening Arrhythmias and Sudden Cardiac Death by SHRs from Univariable Fine and Gray Regression Analyses

**Table 4.**
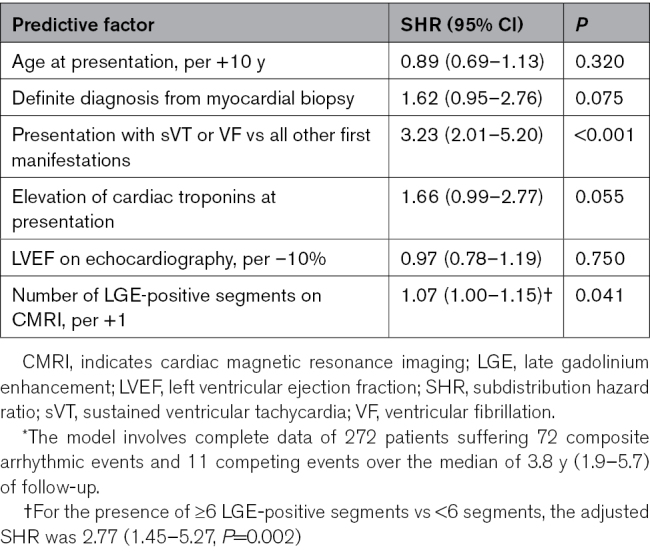
A Fine and Gray Multivariable Model^*^ for the Prediction of Life-Threatening Arrhythmias and Sudden Cardiac Death

### Receiver Operating Characteristic Analyses

**Figure 2.**
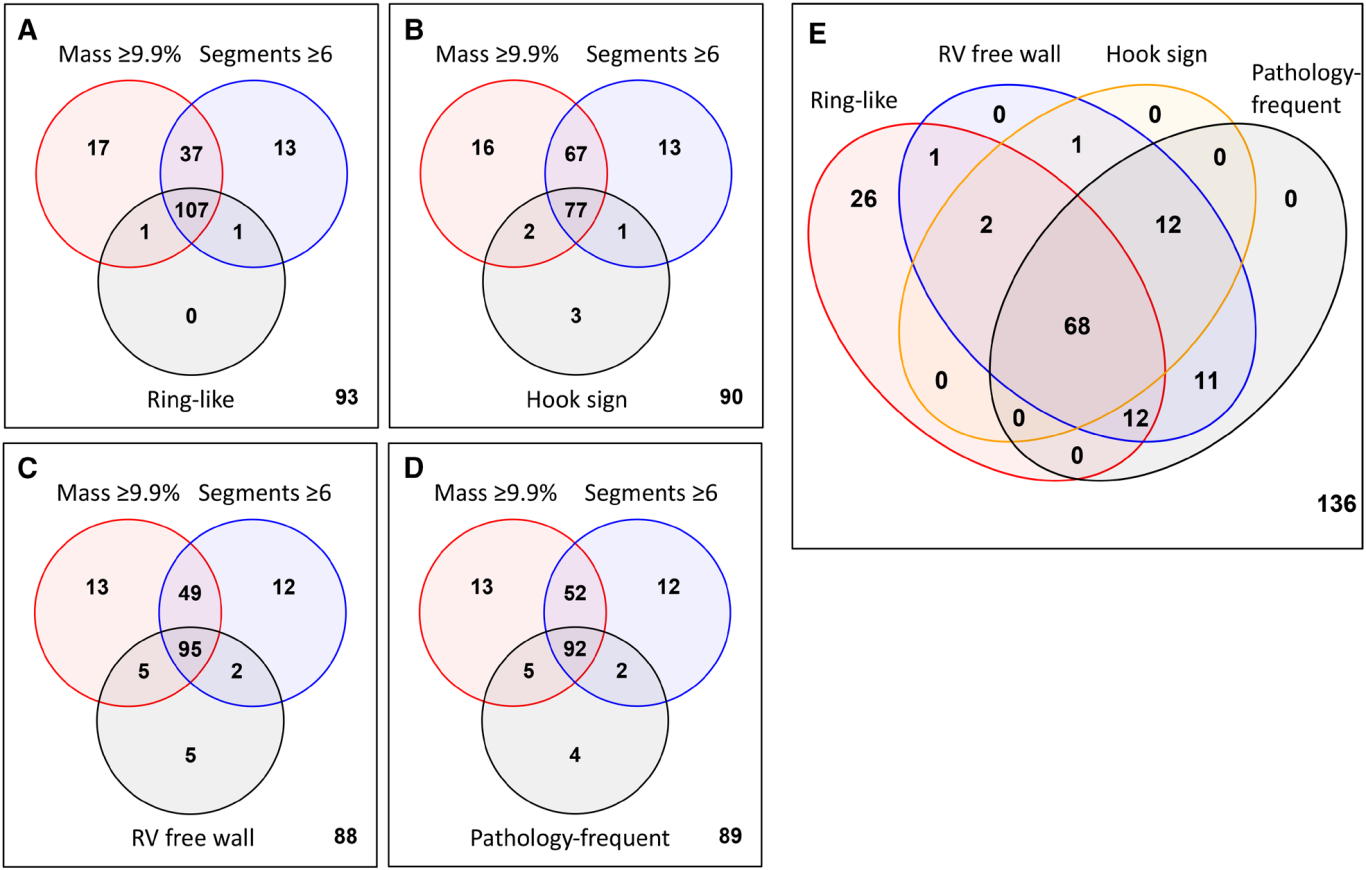
**Overlap across the distribution phenotypes and the extent of late gadolinium enhancement (LGE) in cardiac sarcoidosis.** Venn diagrams demonstrate how patients with (**A**), ring-like LGE; (**B**) the hook sign; (**C**) right ventricular (RV) free-wall LGE; and (**D**) 4 of 4 features of pathology-frequent LGE overlap with groups having ≥6 LGE-positive left ventricular segments out of 17 or an LGE mass ≥9.9%. **E**, Shows the extent of overlap across groups of patients with different LGE imaging phenotypes. Numbers in the lower right corners represent the number of patients outside all circles (groups or phenotypes).

Table 5 summarizes the results of time-dependent receiver operating characteristic analyses of the performance of the LGE variables and LVEF in predicting 2-year and 5-year rates of SCD/VT. The discriminative thresholds, based on the Youden index, were ≥6 for LGE-positive segments, ≥9.9% for the LGE mass, and 4 of 4 for the count of the pathology-frequent LGE criteria. The areas under the curves and the negative predictive values of all CMRI variables decreased from 2 to 5 years of follow-up, indicating a weakening of their predictive capacity. Notably, at 5 years of follow-up, baseline LVEF was no longer statistically significantly discriminative between patients with and without arrhythmic events. The negative predictive values were highest for the LGE mass and the number of LGE-positive segments. However, overall, the differences in the performance metrics across the LGE variables were relatively small (Table 5). Relevant in this respect, there was extensive overlap across the groups of patients testing positive for the different LGE phenotypes (Figure 2A through 2E). Furthermore, aside from 5 individual cases, every patient testing positive for the LGE phenotypes also had either an LGE mass ≥9.9% or ≥6 LGE-positive segments, or both.

**Table 5.**
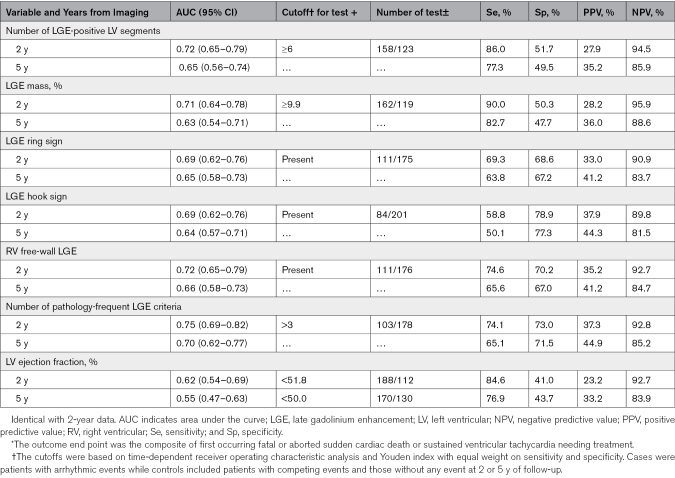
Two-Year and 5-Year Receiver Operating Characteristic Analyses of Variables from Cardiac Magnetic Resonance Imaging as Predictors of Life-Threatening Arrhythmic Events^*^

The incidence graphs and the estimated 5-year and 10-year rates of SCD and SCD/VT stratified by the LGE extent thresholds and the LGE phenotypes are given for the entire cohort in Figures S1 and S2. The 5-year rate of SCD/VT was 11.2% (4.3–18.1%) in patients with LGE mass <9.9% (n=119) versus 37.2% (29.2–45.2%) with higher LGE mass (n=162), and 13.8% (6.4–21.1%) with <6 LGE-positive segments (n=123) versus 36.0% (28.0–44.1%) with ≥6 segments (n=158). The 5-year rate of SCD, in turn, was 4.8% (0.0–9.6%) in patients with LGE mass <9.9% versus 13.7% (8.0–19.5%) for higher LGE mass, and 3.6% (0.0–7.8%) in patients with <6 LGE-positive segments versus 14.9% (8.8–20.9%) with ≥6 segments.

### Cumulative Incidence of SCD/VT by LGE Extent in Patients Without Other ICD Indications in CS

**Figure 3.**
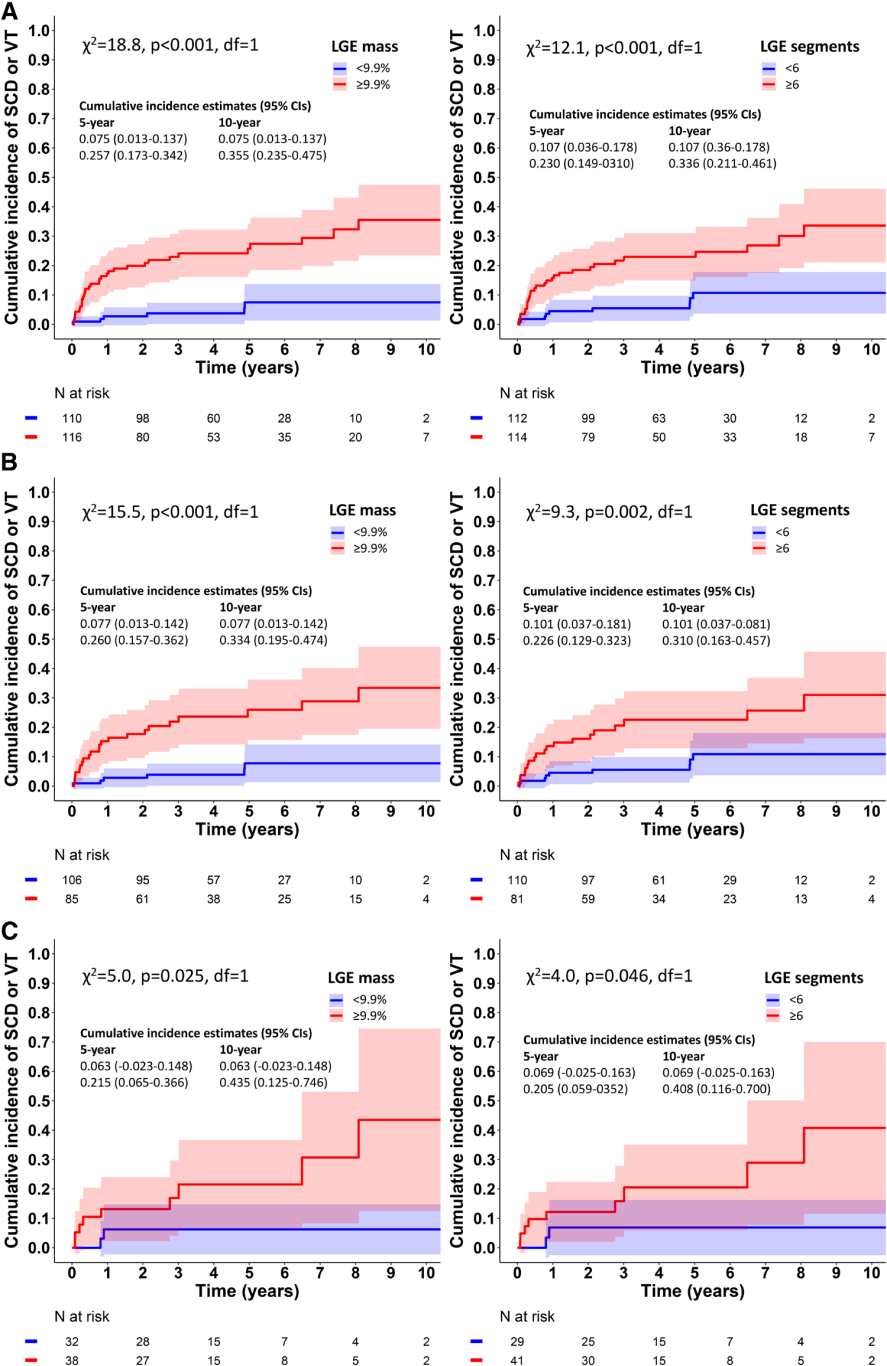
**The composite incidence of sudden cardiac death (SCD) or ventricular tachycardia (VT) in cardiac sarcoidosis by the extent of late gadolinium enhancement (LGE) and the type of cardiac presentation. A**, Shows the incidence of SCD/VT in patients who presented without ventricular tachyarrhythmia and had no indication for a secondary prevention implantable cardioverter defibrillator (ICD; n=226), while (**B**) refers to patients without either ventricular tachyarrhythmia or ejection fraction ≤35% (n=191), and (**C**) represents patients without any current class I or class II ICD indication independent of LGE (n=70). The patients are stratified by the thresholds of LGE mass and the number of LGE-positive segments.

Figure 3A through 3C show the graphs and the numerical estimates for the cumulative incidence of SCD/VT by the thresholds of LGE extent in patients who at presentation were either (1) free of VF and VT (n=226; Figure 3A), (2) free of VT/VF and LVEF ≤35% (n=191; Figure 3B), or (3) free of any current class I or class II ICD indications unrelated to LGE (n=70; Figure 3C). The 5-year incidence of the composite of SCD and VT varied across these subgroups from 6.3% to 7.7% for LGE mass <9.9% and from 6.9% to 10.7% for <6 LGE-positive segments. In all candidates for a primary prevention ICD (n=226, see above), the rate of SCD/VT per 100 patient-years was 1.2 (0.4–2.6) with LGE mass <9.9% versus 6.6 (4.5–9.2) with higher LGE mass and 1.7 (0.8–3.3) with <6 LGE-positive segments versus 6.2 (4.2–8.8) for ≥6 segments. The respective rate of SCD was 0.6 (0.1–1.7) per 100 patient-years for LGE mass <9.9% and 0.6 (0.1–1.6) for <6 LGE segments. The graphs of SCD/VT per 4 contiguous strata of LGE mass and LGE-positive segments, visualizing more closely the dependence of SCD/VT on the dose of LGE, are shown in the Supplemental Material (Figure S3). These graphs show that a small part (10%) of patients without VT/VF at presentation had minimal LGE on CMRI (≤2.5% of LV mass or ≤1 LGE segment) with no SCD/VT events on follow-up.

## Discussion

The accomplishment of the present work was driven by the guidelines from the American College of Cardiology/AHA/HRS^8^ consortium and the ESC^9^ raising CMRI among the tools to help assess the risk of SCD for consideration of ICD implantation in CS. Their class IIa recommendations for an ICD in the presence of stand-alone extensive^8^ or significant^9^ LGE suggest that there is a quantity of LGE, with yet undefined specifics, entailing survival benefit from ICD implantation for primary prevention. Our key finding was that myocardial LGE mass ≥9.9% or the presence of ≥6 LGE-positive LV segments could represent extensive/significant LGE in the prognostic sense, as they were associated with a 5-year rate of SCD close to 15%, while the rate remained <5% with less LGE. Importantly, in the absence of the main class I and class IIa indications for an ICD, the composite 5-year rate of SCD/VT was <7% in patients having LGE mass <9.9% or <6 LGE segments. Other noteworthy observations were that several imaging phenotypes featuring the distribution of LGE, that is, ring-like LGE, the hook sign, RV free-wall LGE, and the count of pathology-frequent LGE features, were predictive of SCD/VT, and that LGE extent appeared to outperform LVEF as a prognostic factor. Finally, all baseline CMRI variables showed a weakening over time in their power to discriminate between patients with and without future arrhythmic events.

### LGE Imaging and the Risk of SCD in CS

In CS, myocardial LGE represents granulomatous scarring and inflammation, delaying contrast washout from the interstitium. It is included as a major criterion in the CS diagnostic schemes^7,26,27^ and commonly presented as a key prognostic factor, too.^8,9,27^ In a comprehensive meta-analysis of 29 CMRI studies involving mainly cohorts of extra-CS and suspected CS, the mere presence of LV LGE had odds ratios of 8.0 (95% CI, 4.3–15.0) for all major cardiac events, and, in 14 studies, 9.0 (95% CI, 4.6–17.5) for ventricular tachyarrhythmias.^28^ Its strong predictive power in extra-CS and in mixed cohorts of suspected and known CS is a matter of course, however, because LGE picks up from the cohort patients with myocardial involvement and an inherently higher risk. In confirmed CS, by contrast, the presence of LGE per se cannot serve as a proper prognosticator due to its constituting a diagnostic criterion and being rarely absent in true CS.^29^ Regarding the quantity of LGE as a risk factor, 2 groups have reported on the association of life-threatening ventricular tachyarrhythmias with the extent of LGE in confirmed as opposed to suspected CS. In a study of 51 patients with CS experiencing 13 events of VT or VF, Crawford et al^30^ found that LGE mass ≥6.0% predicted VT/VF with 75% sensitivity, 82% specificity and an area under the curve of 0.79, while having ≥9 LGE-positive out of 29 LV and RV segments had 92% sensitivity, 88% specificity, and an area under the curve of 0.90 in receiver operating characteristic analysis. Curiously for CS, 19/51 patients did not have LGE at all.^30^ More recently, a retrospective analysis of routine CMRI reports from 298 patients collected from >30 Japanese hospitals suggested that having ≥4 LGE-positive LV segments predicts an increased risk of future SCD and VT.^5^ The event rates were not specified, however, nor was LGE mass studied. The discriminative thresholds of LGE extent found here deviate from the respective thresholds reported by Crawford et al^30^ and Nabeta et al.^5^ However, there are major differences between these studies^5,30^ and ours in ethnic background, criteria for CS diagnosis, proportions of histologically proven and definite CS, and whether the LGE data were based on a centralized and blinded analysis, as in our study, or extracted from routine reports, as in the Japanese work.^5^

Of the specific LGE imaging phenotypes analyzed here, the hook sign has been raised as a signature imaging biomarker of CS,^19,31^ while RV free-wall LGE and the count of the pathology-frequent LGE features have been identified as prognostic signs in suspected CS.^20,32^ The ring-like LGE, in turn, has portended poor outcomes in nonischemic and inflammatory cardiomyopathies.^18,33^ The phenotypes and the count of pathology-frequent LGE features predicted future SCD/VT in our cohort due, in all likelihood, to their strong link with the extent of LV LGE (Figure 2). Due to their mutual overlap, the phenotypes had rather similar predictive capacities (Table 5; Figure S2). It has been reported that RV free-wall LGE predicts ventricular tachyarrhythmias even independent of the extent of LV LGE in patients with extra-CS.^32,34^ Due to the nearly complete overlap of RV free-wall LGE with extensive LV LGE (Figure 2), such an association could not be validated in our cohort of confirmed CS. Regardless, we believe that the LGE phenotypes, being readily recognizable, can serve as red flags alerting the observer to the possibility of extensive LV scarring and a predisposition to fatal arrhythmias.

### Clinical Implications

Our work adds nuances into the stratification of the risk of SCD in CS. Currently, 3 guidelines from the Western cardiology societies^7–9^ help clinicians and patients weigh the benefits and risks of an ICD in CS. Their recommendations are copied unmodified in the most recent American and European consensus statements on the management of CS.^27,35^ The guidelines share many recommendations but are divided on 2 clinically important aspects. First, the 2014 HRS statement^7^ requires a period of immunosupression (if there is active inflammation) before ICD implantation for severe LV dysfunction (LVEF <35%), while the 2017 American College of Cardiology/AHA/HRS guideline,^8^ by contrast, emphasizes rapid therapeutic interventions because immunosuppression is unlikely to revert advanced LV dysfunction. The ESC guideline^9^ does not require preimplantation immunosuppression either. Second, and more pertinent to our theme, presence of myocardial LGE serves as an independent class IIa indication for an ICD in the 2017 American College of Cardiology/AHA/HRS^8^ and the 2022 ESC guidelines,^9^ but not in the 2014 HRS statement,^7^ where it constitutes an indication for an electrophysiological study. Our findings support the former recommendation, adding that a more nuanced definition of high-risk LGE than just extensive or significant is feasible.

In CS, ICD indications have not been discussed per categories of estimated SCD risk. In hypertrophic cardiomyopathy, current guidelines for prevention of SCD from ESC^9,36^ give a class IIa recommendation for an ICD if the 5-year risk estimate^37^ is ≥6% for SCD and its equivalents (including appropriate ICD shocks but not antitachycardia pacing). In clinically manifest cardiomyopathies due to *LMNA* mutations, the ESC guideline^9^ recommends an ICD (class IIa) if the 5-year risk estimate^38^ for SCD and life-threatening ventricular tachyarrhythmia (VT converted by ICD therapy or causing hemodynamic instability) is ≥10%. Laminopathies and CS share several clinical manifestations including impaired conduction, progression to end-stage heart failure, and a high risk of SCD.^9,36^ From the perspective of these recommendations, the present data (Figure 3) suggest to us that, in patients who have no other guideline-based class I or IIa ICD indications, implantation could be considered if LGE mass is ≥9.9% by the full-width at half-maximum method or the LGE involves ≥6 LV segments. Less extensive LGE predicted a 5-year SCD/VT rate <7% with a SCD risk of 0.6 per 100 patient-years, and either electrophysiological study^7,9^ or close surveillance with repeat risk assessments could be discussed at shared decision-making. The risk of SCD/VT relative to a wider range of LGE extents than just dichotomy should also be taken up, and patients with CS having small quantities of LGE, for example, ≤2.5% of LV mass or in only 1 LV segment, should be informed of their minimal risk of SCD (Figure S3). The present threshold for high-risk LGE mass, 9.9%, is supported by a recent follow-up study where a myocardial PET perfusion defect exceeding 10% of LV mass was the most significant prognostic factor in 113 patients with events dominated by ventricular tachyarrhythmias.^39^ Yet, it is self-evident that decisions regarding ICD implantations cannot rely blindly on decimals of LGE mass or LGE segment counts. Issues involving imaging and image analysis can introduce variation across institutions and readers, and an element of uncertainty needs to be factored in decision-making. Ultimately, all decisions should be shared with patients in consideration of their age, general health, competing mortality risk, possible device complications, and the impact of an intracardiac device on lifestyle and psychological health.

### Strength and Limitations

The uniqueness of our work is that we were able to analyze the prognostic value of CMRI in a large cohort of biopsy-proven CS with nearly half of the patients having a definite diagnosis from myocardial histology. Accordingly, the predictive performance of LGE imaging reported here pertains to confirmed CS as opposed to most past reports involving extra-CS with suspected cardiac involvement. Our findings do not cover subclinical CS, however, because enrollment in the underlying MIDFIN registry does not include systematic cardiac screening of patients with known sarcoidosis. The lack of screening also explains why less than one fifth of our cohort had a history of sarcoidosis on admission, the others having de novo CS. The present findings may not be directly generalizable to diverse ethnic groups either, because our study only involved a White population of Northern European ancestry. Due to the retrospective design and the long coverage of our work, the diagnostic use of CMRI in myocardial diseases, as well as the scanners and imaging methods, evolved over time and varied across the participating hospitals. Quality issues resulted in the rejection of 5% to 10% of available studies, depending on the modality. The image analyses were made by a single expert only, but both the intraobserver and interobserver repeatability of the data were acceptable. There exist several somewhat different techniques to assess LV LGE mass on CMRI,^15,16^ and the 9.9% cutoff for high risk found here is directly applicable only for the full-width at half-maximum method. Although our cohort was large for a CMRI study in biopsy-proven CS, some subgroups were small and had moderately few SCD/VT events resulting in wide 95% CIs for the event rates. The cut offs for LGE extent (LGE mass and segments) were not determined a priori. Due to the rarity of CS, we were not able to confirm the discriminative cut offs in an independent CS cohort and, pending validation, they should be considered only suggestive in clinical work. The statistical tests replacing the count of LGE-positive segments in the multivariable model (Table 4) with other LGE variables, 1 at a time, were not adjusted to account for multiple testing; they were considered secondary analyses. Finally, the extent of diffuse interstitial fibrosis, quantifiable by the extracellular volume fraction on CMRI,^40^ could not be assessed in our work. Its prognostic role should be studied in future, although, compared with patchy myocardial scarring, interstitial fibrosis is expected to be less arrhythmogenic.

### Conclusions

Clinically manifest CS is characterized by a dose-response relationship between the extent of LGE on CMRI and the risk of SCD and life-threatening arrhythmias. Our analyses raise LGE mass ≥9.9% by the full-width at half-maximum method or presence of LGE in at least 6 of 17 LV segments as statistically optimized indicators for high risk in histologically confirmed CS. Subject to quality imaging and carefully standardized analysis, patients with less LGE and no established ICD indications may be expected to have a SCD risk of 0.6/100 patient-years and a <7% composite risk of SCD or VT in 5 years of follow-up. These thresholds of LGE extent found here may help objectify what prognostically extensive/significant LGE is when discussing implantation of a primary prevention ICD in CS. However, their prospective validation is needed.

## ARTICLE INFORMATION

### Acknowledgments

The authors thank our colleagues and staff in all participating hospitals for help with this study.

### Sources of Funding

Dr Pöyhönen was supported by the Finnish Cultural Foundation (Helsinki, Finland), Finnish Foundation for Cardiovascular Research (Helsinki, Finland) and Finnish government grant for medical research (Helsinki, Finland).

### Disclosures

Valtteri Uusitalo, Scientific collaboration and lecture fee with GE Healthcare and lecture fee and advisory board activity with Pfizer. The other authors report no conflicts of interest.

### Supplemental Material

Figures S1–S3

## Supplementary Material


