# Supplementary material for: Magnetic Resonance Imaging in the Assessment of the Risk of Sudden Death in Cardiac Sarcoidosis: What Is Extensive or Significant Late Gadolinium Enhancement?
Source: Circ Arrhythm Electrophysiol. 2024 Dec 20;18(1):e013239. doi: 10.1161/CIRCEP.124.013239 (PMC11753451; doi:10.1161/CIRCEP.124.013239)
Supplement: Supplementary file 1 [file hae-18-e013239-s001.pdf]

SUPPLEMENTAL MATERIAL

Supplemental Figures

Figure S1 (A-D).

Figure S2 (A-H).

Figure S3 (A-B).

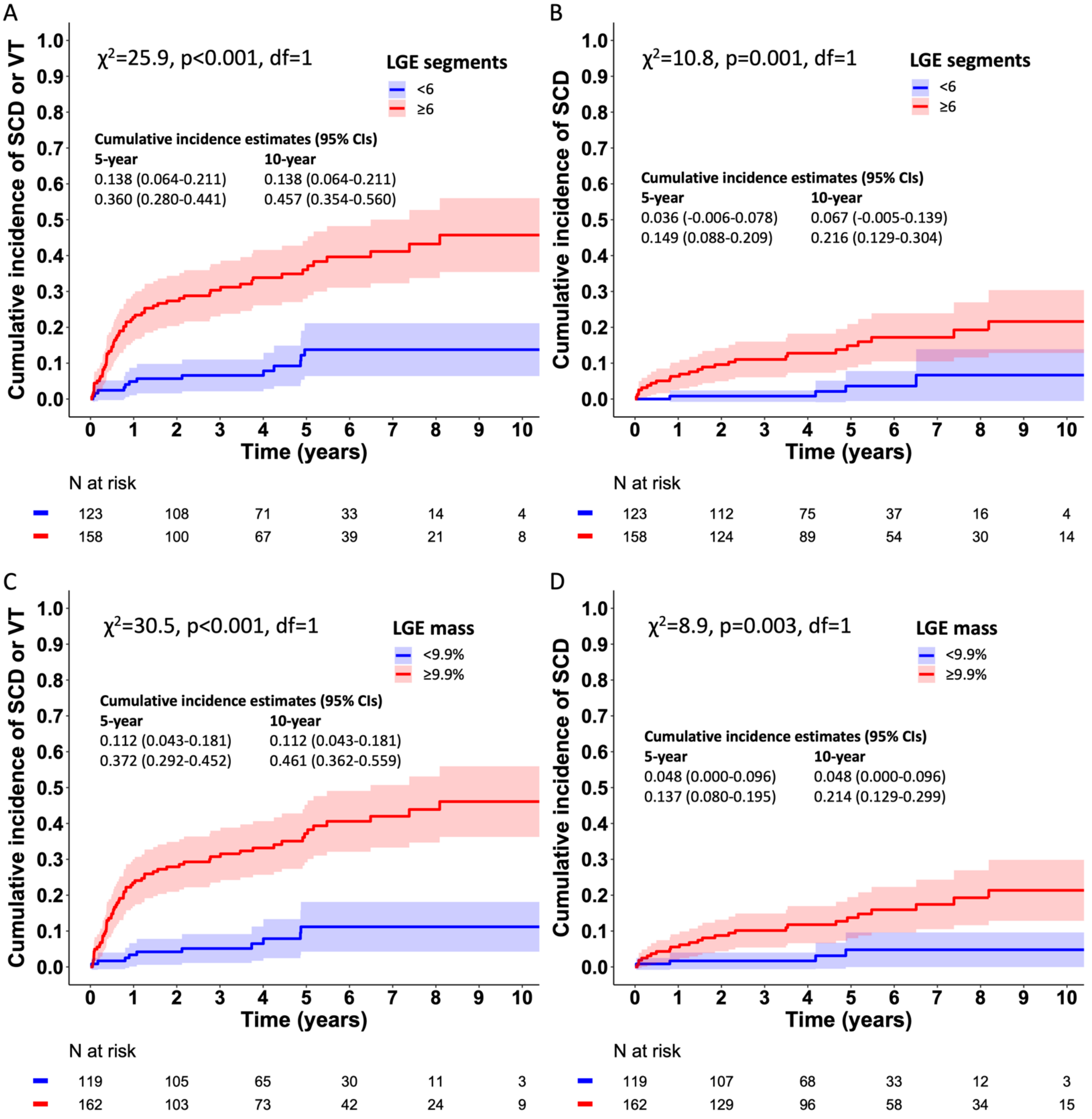

Figure S1. Cause-specific cumulative incidence graphs for the composite of sudden cardiac death (SCD) and sustained ventricular tachycardia (VT) (panels **A** and **C**) and SCD alone (panels **B** and **D**) in patients with cardiac sarcoidosis stratified by the number of left ventricular segments with late gadolinium enhancement (LGE) (panels **A** and **B**) and LGE mass (panels **C** and **D**).

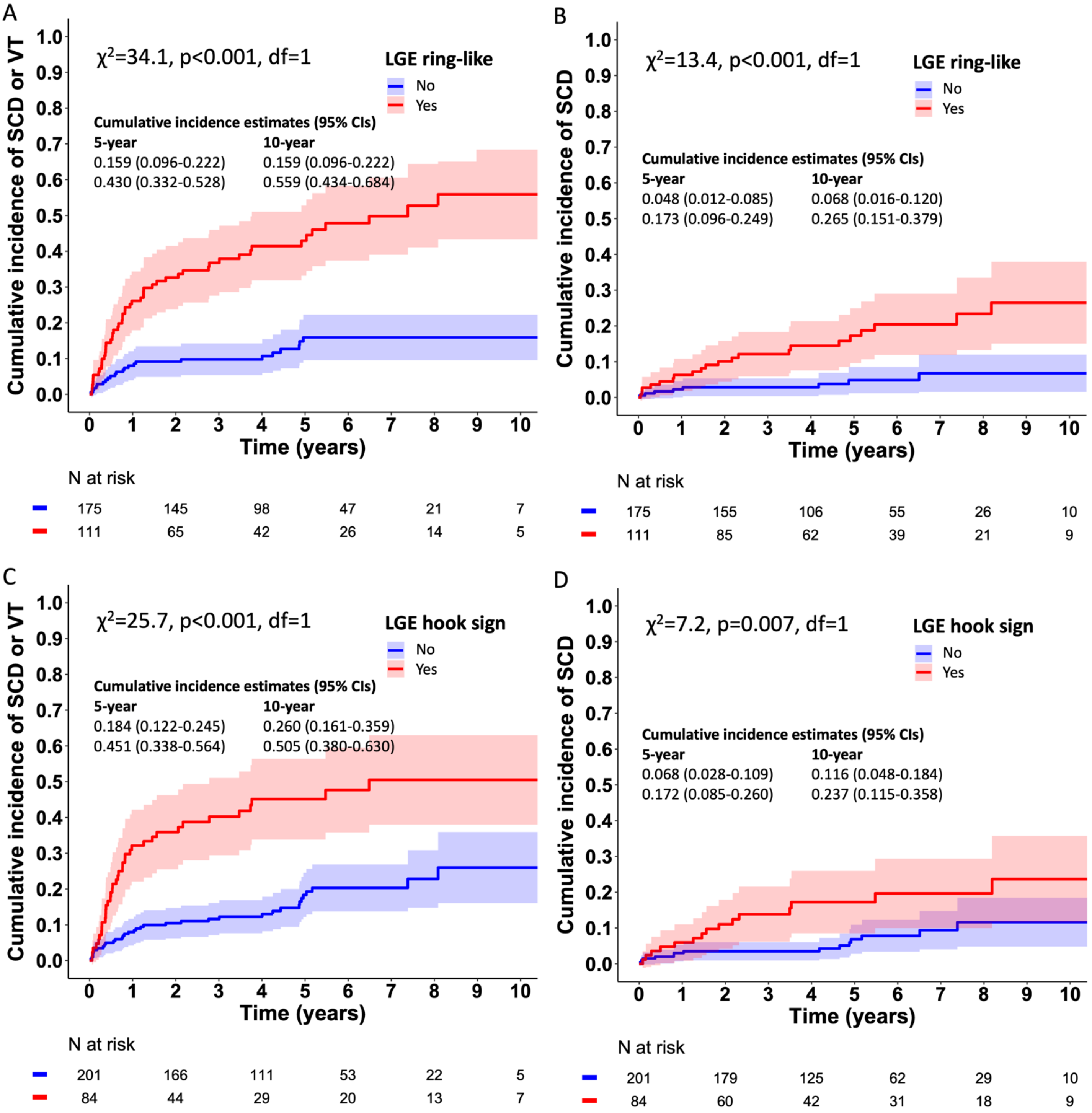
A
χ2=34.1, p<0.001, df=1
LGE ring-like
No
Yes
Cumulative incidence estimates (95% CIs)
5-year
0.159 (0.096-0.222)
0.430 (0.332-0.528)
10-year
0.159 (0.096-0.222)
0.559 (0.434-0.684)
Cumulative incidence of SCD or VT
Time (years)
N at risk
175 145 98 47 21 7
111 65 42 26 14 5
B
χ2=13.4, p<0.001, df=1
LGE ring-like
No
Yes
Cumulative incidence estimates (95% CIs)
5-year
0.048 (0.012-0.085)
0.173 (0.096-0.249)
10-year
0.068 (0.016-0.120)
0.265 (0.151-0.379)
Cumulative incidence of SCD
Time (years)
N at risk
175 155 106 55 26 10
111 85 62 39 21 9
C
χ2=25.7, p<0.001, df=1
LGE hook sign
No
Yes
Cumulative incidence estimates (95% CIs)
5-year
0.184 (0.122-0.245)
0.451 (0.338-0.564)
10-year
0.260 (0.161-0.359)
0.505 (0.380-0.630)
Cumulative incidence of SCD or VT
Time (years)
N at risk
201 166 111 53 22 5
84 44 29 20 13 7
D
χ2=7.2, p=0.007, df=1
LGE hook sign
No
Yes
Cumulative incidence estimates (95% CIs)
5-year
0.068 (0.028-0.109)
0.172 (0.085-0.260)
10-year
0.116 (0.048-0.184)
0.237 (0.115-0.358)
Cumulative incidence of SCD
Time (years)
N at risk
201 179 125 62 29 10
84 60 42 31 18 9

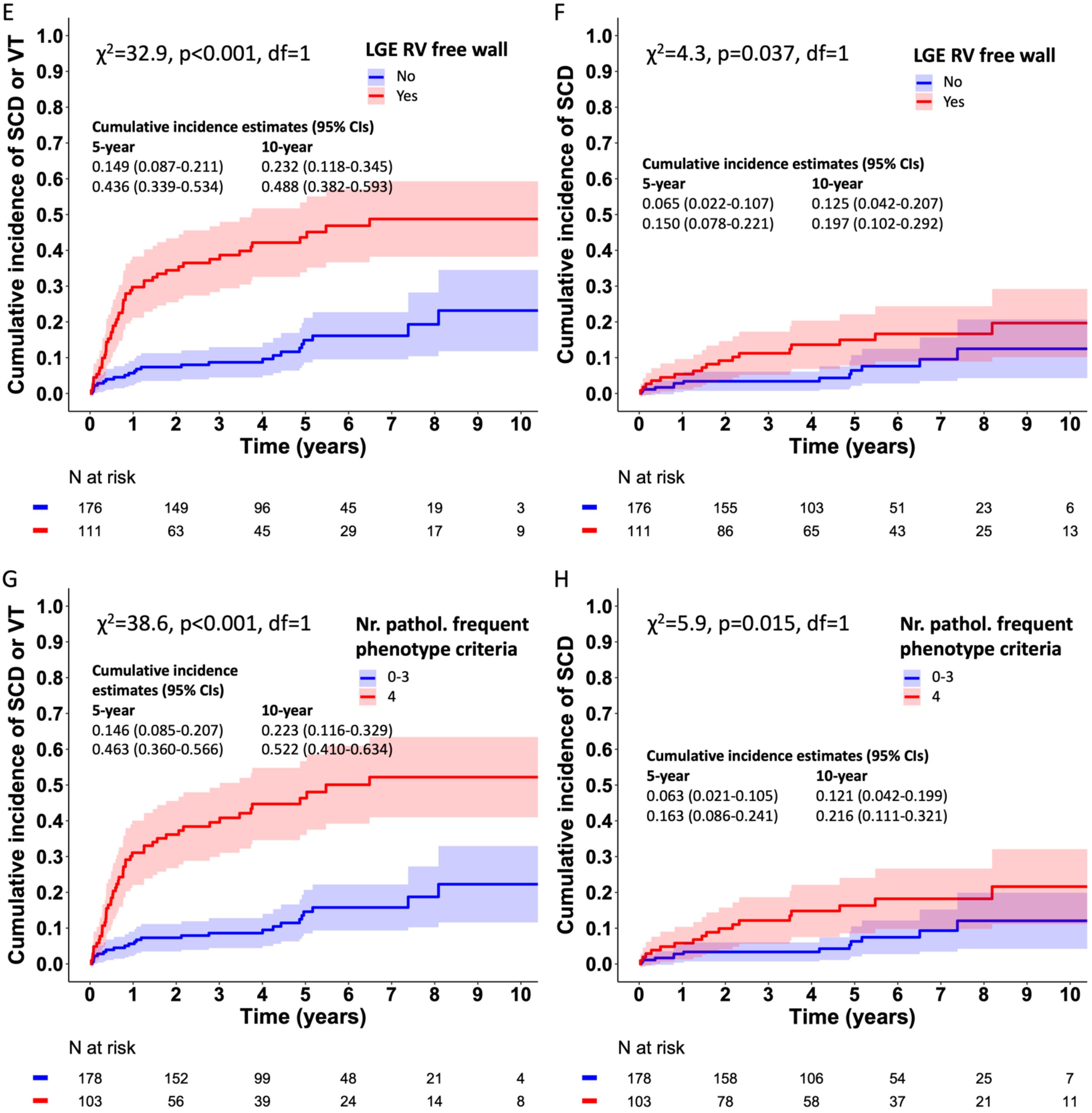

Figure S2. Cause-specific cumulative incidence graphs for the composite of sudden cardiac death (SCD) and sustained ventricular tachycardia (VT), and SCD alone, in patients with clinically manifest cardiac sarcoidosis stratified by presence of (**A, B**) ring-like late gadolinium enhancement (LGE); (**C, D**) LGE hook sign; (**E, F**) right ventricular (RV) free wall LGE; and (**G, H**) the number of pathology-frequent phenotype criteria.

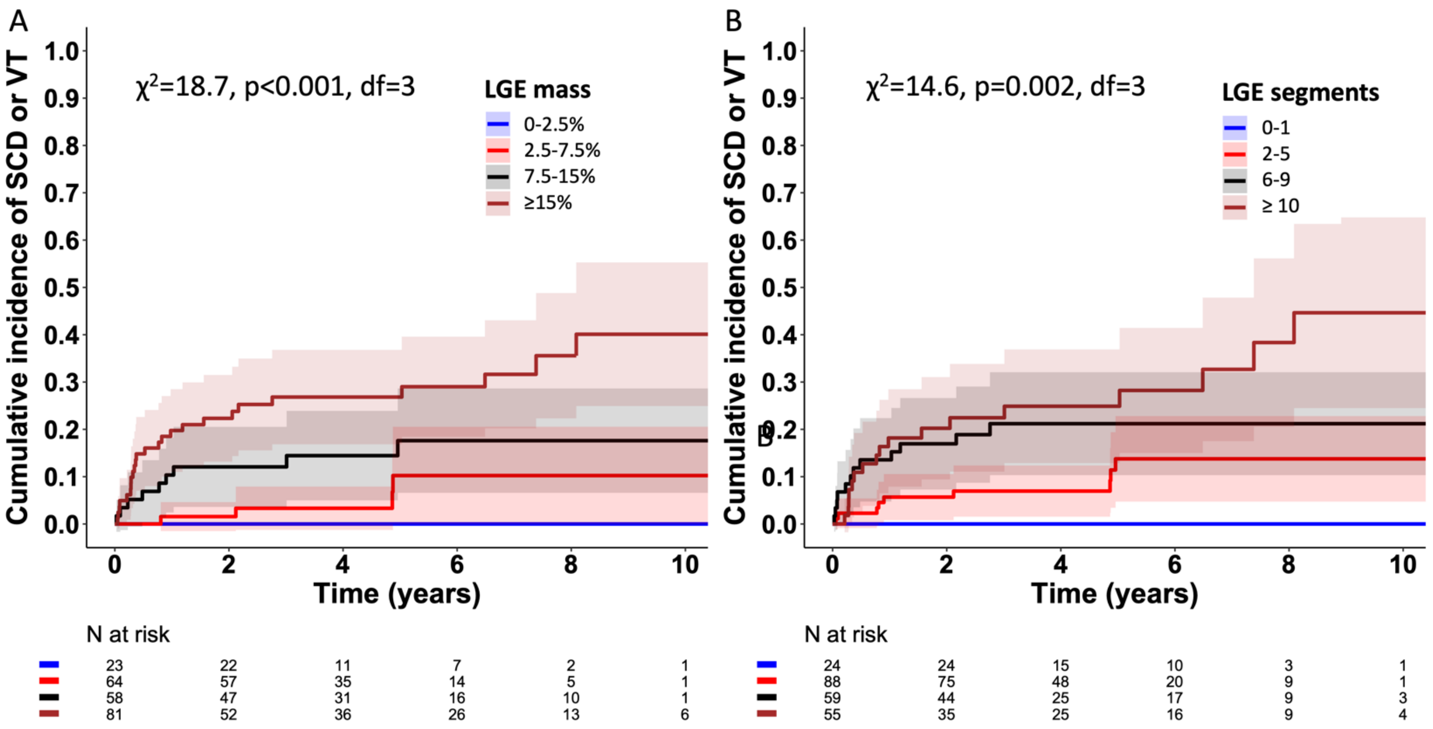

Figure S3. Cause-specific cumulative incidence graphs for the composite of sudden cardiac death (SCD) and sustained ventricular tachycardia (VT) in patients with clinically manifest cardiac sarcoidosis and no ventricular fibrillation or tachycardia at presentation (n=226) stratified by left ventricular late gadolinium enhancement (LGE) mass (**A**) and the number of LGE-positive segments out of 17 (**B**).
